# The Using of Nanoparticles of Microalgae in Remediation of Toxic Dye from Industrial Wastewater: Kinetic and Isotherm Studies

**DOI:** 10.3390/ma15113922

**Published:** 2022-05-31

**Authors:** Abdallah Tageldein Mansour, Ahmed E. Alprol, Khamael M. Abualnaja, Hossam S. El-Beltagi, Khaled M. A. Ramadan, Mohamed Ashour

**Affiliations:** 1 Animal and Fish Production Department, College of Agricultural and Food Sciences, King Faisal University, P.O. Box 420, Al-Ahsa 31982, Saudi Arabia; 2 Fish and Animal Production Department, Faculty of Agriculture (Saba Basha), Alexandria University, Alexandria 21531, Egypt; 3 National Institute of Oceanography and Fisheries (NIOF), Cairo 11516, Egypt; 4 Department of Chemistry, College of Science, Taif University, P.O. Box 11099, Taif 21944, Saudi Arabia; k.ala@tu.edu.sa; 5 Agricultural Biotechnology Department, College of Agriculture and Food Sciences, King Faisal University, P.O. Box 420, Al-Ahsa 31982, Saudi Arabia; helbeltagi@kfu.edu.sa; 6 Biochemistry Department, Faculty of Agriculture, Cairo University, Giza 12613, Egypt; 7 Central Laboratories, Department of Chemistry, King Faisal University, P.O. Box 420, Al-Ahsa 31982, Saudi Arabia; kramadan@kfu.edu.sa; 8 Department of Biochemistry, Faculty of Agriculture, Ain Shams University, Cairo 11566, Egypt

**Keywords:** *Arthrospira platensis* NIOF17/003, bioremediation, green nanoparticles, methylene blue, adsorption, equilibrium isotherm, SEM, FTIR

## Abstract

Batch adsorption experiments were carried out to study the removal of the toxic Methylene Blue Dye (MBD) from synthetic aqueous solutions using the nanoparticles form of *Arthrospira platensis* NIOF17/003. The adsorption capacity of the adsorbent for MBD was investigated using different amounts of *A. platensis* nanoparticles at different contact times, temperatures, pH, and MBD initial concentrations in the synthetic aqueous solution. In addition, *A. platensis* nanoparticles were characterized using Electron Microscopy (SEM), Brunauer–Emmett–Teller (BET), Fourier Transform Infrared (FTIR), and Ultraviolet spectra (UV) techniques. The optimum removal of MBD was found at a concentration of 0.4 g *A. platensis* nanoparticles. *A. platensis* nanoparticles remove 93% of MBD in 5 min (under agitation conditions at 150 rpm). The highest adsorption capacity was found by the Langmuir model to be 58.8 mg g^−1^. It is an endothermic process with spontaneity increasing with temperature. The probable mechanism for the adsorption is chemisorption via surface-active charges in the initial phase, which is followed by physical sorption by occupying pores of *A. platensis*. MBD adsorption by *A. platensis* follows pseudo-second-order kinetics. The Freundlich and Langmuir models fit well with the experimental data. The adsorption experiments suggested that the regeneration of the adsorbents was possible for repeated use, especially regarding MBD up to 65.8% after three cycles, which proves it can be easily recycled. In conclusion, the nanoparticles of *A. platensis* have a significant adsorption potential in the removal of MBD from effluent wastewater.

## 1. Introduction

Green techniques of nanomaterial synthesis are part of sustainable chemistry approaches. The utilization of renewable raw materials as preparations for the reduction in metal ions to nanoparticles (NPs) has recently piqued interest. Synthetic dyes are polluting chemicals that have both harmful and aesthetic effects on aquatic habitats. Dye effluents, which contain dyed organic molecules, increase the organic load in water bodies and reduce sunlight penetration, reducing phytoplankton photosynthetic activity and altering the biological balance of the aquatic ecosystem [1]. Dyes are significant dangerous materials used in several industrial sectors. Methylene Blue Dye (MBD) is a cationic dye that has been used to color cotton, wool, and silk for centuries [2]. Breathing difficulties, nausea, vomiting, tissue necrosis, intense sweating, mental confusion, cyanosis, and metabolic disturbances are all side effects of MBD [3]. This dye has a harmful impact on the water ecosystem and its components [4,5]. MBD has been extensively used in adsorption experiments, including low-cost adsorbents, such as marine algae [6], fungi [7], activated carbon [8,9], agricultural [10], and industrial wastes [11,12].

Many physical, chemical, and biological approaches [13,14] have been investigated for the treatment of synthetic dye-contaminated wastewaters [15], such as chemical coagulation/flocculation [16,17], chemical precipitation [18], oxidation processes [19], ion exchange [20], ultra-filtration [21], and reverse osmosis [22]. Each of these methods has disadvantages, such as high operational and capital costs, low efficiency at low dye concentrations, and the creation of unwanted sludge [15]. Because these synthetic dyes resist microbial degradation, traditional biological wastewater treatment methods have grown less effective in removing them [23]. For these reasons, nanoparticles could be used to treat wastewater as they have a high specific surface area, which improves dye removal activity [24]. Accordingly, to decrease dye concentration in wastewater, it is vital to create effective, low-cost, and environmentally friendly solutions [25]. Furthermore, previous chemical and physical techniques used hazardous materials, required high conditions such as temperature, energy, and pressure, and produced hazardous by-products. As a result, interest in biological techniques or green nanotechnology has grown [26]. Green nanotechnology refers to a method for producing nanomaterials that is free of or uses less harmful components throughout the creation process. Biological elements such as bacteria, actinomycetes, fungus, cyanobacteria, macro-algae, and plants can all be used to green synthesize NPs. Because of its eco-friendliness, cost-effectiveness, ease of handling, upscaling, and biocompatibility, green synthesis is favored over chemical and physical approaches [27]. Several NPs have recently been produced using environmentally friendly technologies. Adsorption is quickly gaining prominence among treatment technologies, with the realization that it may provide high-quality water while being a cost-effective technique [28,29]. However, microalgae and cyanobacteria (formerly known as blue-green algae) have been identified as potential organisms for heavy metal removal, dye removal from industrial effluents, pollutant bioremediation, and the production of commercially important molecules [30,31,32,33].

Many aquatic plant biomasses, such as microalgae and seaweeds, have been utilized as renewable, biological, and high-efficiency absorption materials [34,35,36,37,38,39,40]. In general, algal cells are one of the most promising aquatic organisms that contain biomolecules, playing a vital role in several biological and sustainable industries such as biofertilizers [41,42,43], aquaculture [41,44,45], bioenergy [46,47,48,49], antioxidant and antimicrobial materials [50,51,52], and pharmaceuticals [53], besides being effective, sustainable, and low-cost effective adsorption biomaterials [32,47,54,55,56,57].

The blue-green algae *Arthrospira platensis* is a widely cultivated species around the world and is available in large quantities, reaching 2000 tons per year [53,58,59]. *Arthrospira platensis* is a filamentous cyanobacterium with a number of benefits, including quick growth rates, high biomass output, ease of cell harvesting, and biomass composition manipulation [48,60,61], and it is a possible biosorbent [32,33]. The cyanobacterium *Arthrospira platensis* is known to have a good supply of protein, polysaccharides, lipids, minerals, vitamins, and phosphate [48,60,61], all of which are required for dye binding [32,33,62,63]. According to Seker et al. [64], several functional groups in blue-green algal biomass, including carboxyl, hydroxyl, sulfate, phosphate, and other charged groups, may be responsible for dye binding. This cyanobacterium is widely available, and it can be used to adsorb heavy metals such as cadmium, copper, lead, and nickel [23].

The use of nanoparticles from biomass could be an effective technique to boost the bio-available mass’s adsorption sites [65]. Furthermore, recent breakthroughs in nanotechnology have demonstrated that ultra-fine adsorbents are a viable alternative to dye removal [66].

There are several forms of cyanobacteria species *A. platensis* (live form, biomass form, dried form, and nanoparticles form), which can be utilized as adsorbents materials of the cationic dye, MBD. However, exposure of the live form of *A. platensis* to MBD increased the toxicity, reduced growth rate, pigment synthesis, and protein content, due to dye uptake by the live form of *A. platensis* [67]. The only study using *A. platensis* that is linked to this purpose is an artificial neural network to estimate methylene blue adsorption capacities onto *Spirulina* sp. [54]. There is no published data on the adsorption of cationic dyes on the nanoparticle form of *A. platensis*. However, there is little information in the literature about the adsorptive kinetics and thermodynamics of MBD onto *A. platensis* or the role of the ion exchange process in dye removal. Even though the ion exchange mechanism plays a key role in MBD removal by various adsorbents, it has not been extensively explored by detection methods. Accordingly, the aim of this study is to examine the adsorption of basic dye methylene blue dye (MBD) from aqueous solutions as a low-cost adsorbent. Effects of different parameters such as pH, initial MBD concentration, adsorbent dose, contact time, and temperature were examined by a batch process to better understand the adsorption rate and processes of MBD onto nanoparticles of *A. platensis*. The kinetics, isotherms, and thermodynamic factors were also calculated to determine the rate constants and adsorption mechanism. Additionally, Scanning Electron Microscopy (SEM), Brunauer–Emmett–Teller (BET), Fourier Transform Infrared (FTIR), and Ultraviolet spectra (UV) were applied to characterize the physicochemical and morphological properties of the adsorbent.

## 2. Materials and Methods

### 2.1. Chemicals and Preparation of Dye Solution

**Figure 1:**
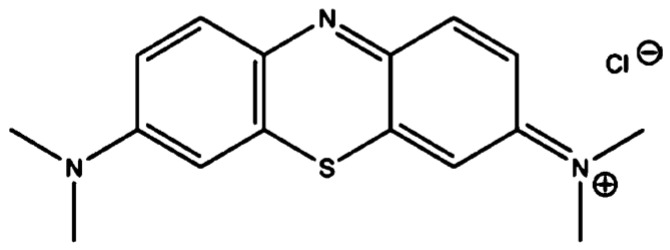
Chemical structure of MBD.

Analytical grade MB supplied by Sigma-Aldrich is utilized as adsorbate without additional purification. MBD is a cationic dye with the chemical formula C_16_H_18_N_3_SCl (Figure 1), a maximum wavelength of 665 nm, and a molecular weight of 319.85 (g mol^−1^). MB dye has strong water solubility and a positive charge on the S atom at 293 K. Melting 1 g of powder dye in one liter of distilled water yielded 1000 mg L^−1^ MB stock solutions. The stock solution was made into working solutions ranging from 5 to 40 mg L^−1^ [68]. Following Beer’s law, low concentrations were utilized to ensure a linear connection between absorbance and concentration. Using an SQ-2800 ultraviolet-visible spectrophotometer, a calibration curve (absorbance against concentration).

### 2.2. Adsorbent Nanoparticles (A. platensis) Preparation

The nanoparticle preparation of *A. platensis* was performed at the Egyptian Petroleum Research Institute (EPRI), Nasr City, Cairo, Egypt, using ball grinding (Planetary Ball Mill PM 400 “4 grinding stations”), as described in our previous work [60]. As our previously published works show [60,61], the distribution of *A. platensis* nanoparticle size was determined by dynamic light scattering (DLS) and recorded with the average sizes of 183.9 nm (87.6%) and 1069 nm (12.4%), while the normal size of the alga *A. Platensis* was 100 μmL [60,61].

### 2.3. Characterization of the Adsorbent (A. platensis Nanoparticle)

#### 2.3.1. Scanning Electron Microscopy (SEM)

SEM (JEOLGSM-6610LV) at an accelerated voltage of 25 kV was used to examine the surface morphology of *A. platensis* nanoparticles. For measurement, the surfaces of the adsorbent were vacuum-coated with gold.

#### 2.3.2. BET Analysis

The NLDFT Ads Model was used to conduct nitrogen (N_2_) adsorption–desorption studies at liquid nitrogen temperature. The specific surface area was estimated using the Brunauer–Emmett–Teller (BET) method by (Micromeritics Instrument Corporation, Model-3Flex, Norcross, GA, USA) using nitrogen adsorption at 77 K using critical pressure 33.5 atm.

#### 2.3.3. FTIR Spectral Analysis

The chemical content of the produced *A. platensis* nanoparticles biomass was determined using the KBr pellet technique and an FTIR (TENSOR Series FT-IR Spectrophotometer, Germany) in the scope of 500–4000 cm^−^^1^.

#### 2.3.4. Ultraviolet Spectra (UV)

As a basic analytical technique, UV–vis spectroscopy was used to evaluate the nano *A. platensis* powdered extracts as well as to determine secondary metabolites. The powder dilution was made in 50 mL volumetric flasks with 0.2 g dissolved in deionized water.

### 2.4. Batch Experiments of the Adsorption Process

All batch adsorption experiments were completed in 250 mL Erlenmeyer flasks containing dry biomass and 50 mL dye solutions. To ensure that the flasks reached equilibrium, they were shaken at 150 rpm for 180 min. The effects of the sorption factors of adsorbent dosage (0.05, 0.1, 0.2, 0.4, and 0.8 g L^−1^) in addition to experimental parameters of initial MBD concentrations (5, 10, 15, 20, and 40 mg L^−1^), contact times (10, 20, 30, 40, 60, 90, 120, and 180 min.), pH values (2, 4, 6, 8, 10, and 12), and temperatures (303, 313, 323, and 333 K) were carried out.

### 2.5. Dyes Removal Efficiency

The amount of dyes absorbed per gram (mg g^−1^) of *A. platensis* nanoparticles may be determined by using the following equation [28] at equilibrium:

q_e_ = [((C_i_ − C_e_) × V)/M] (1)

The percentage of dyes removed (efficiency) obtained by the following equation [69] can also be used to show dye uptake:

(2) $$\mathrm{Adsorption}\;(\%)=[(C_{i}-C_{e})/C_{i}]\times 100$$

where C_i_ is the initial concentration of methylene blue dye, C_e_ is the equilibrium concentration of methylene blue dye (mg L^−^^1^), m (g) is the weight of *A. platensis* biomass, and V (L) is the volume of the methylene blue solution, respectively.

### 2.6. Mathematical Models (Isotherm Study)

The sorption capacity residual concentration and the adsorbate fixed temperature are determined by the adsorption isotherm. Batch sorption experiments were conducted at 303 K of temperature by mixing 0.1 g of adsorbent with 50 mL of a solution of initial MB dye concentration at (5, 10, 20, 30, and 40 mg L^−1^) for 3 h at pH 6; then, these were shaken at 160 rpm of agitation rate. Then, we analyzed the reaction mixture for residual MBD content. Langmuir [70], Freundlich [71], and Tempkin isotherm [72] models (Equations (3)–(5), respectively) were used to analyze the adsorption parameters:

(3) $$q_{e}=\frac{Q_{m}K_{a}C_{e}}{1+K_{a}C_{e}}$$

(4) $$q_{e}=K_{F}C_{e}^{1/n}$$

Q_e_ = B ln A + B ln C_e_ (5)

where Q_m_ is the solute’s highest adsorption capacity (mg g^−^^1^) and K_a_ is the sorption equilibrium constant (L mg^−^^1^), which is connected to Langmuir’s apparent energy of adsorption. K_F_ is the Freundlich constant revealing the comparative sorption capacity of the adsorbent material correlated to the bonding energy. A (L g^−^^1^) = Tempkin isotherm constant also, called equilibrium binding constant, B = (RT)/b, R = gas constant (8.314 J mol^−^^1^ k), T (k) = absolute temperature, the constant (b) is related to the heat of adsorption.

### 2.7. Adsorption Kinetics

At pH 8, the kinetic investigations were carried out using a similar approach. First, 0.1 g of adsorbent was mixed individually with 50 mL of MBD solution (10 mg L^−^^1^ concentrations), and the mixture was obtained at 298 K temperature for the required time intervals of 10, 15, 30, 120, and 180 min [73]. The concentration of MBD in the clear solutions was evaluated.

### 2.8. Theoretic Background of Adsorption Kinetics

#### 2.8.1. Kinetic Model of Pseudo-First-Order

The linear form of the generalized pseudo-first-order equation is represented by the equation below [74]:

dq_t_/dt= K_1_ (q_e_ − q_t_) (6)

where: q_e_ stands for the amount of dyes adsorbed at equilibrium (mg g^−^^1^), q_t_ stands for the amount of dyes adsorbed at time t (mg g^−^^1^), and K_1_ stands or the pseudo-first-order rate constant (min^−^^1^). Below are the steps used to calculate the integrating equation:

log (q_e_/q_e_ − q_t_) = k_1_t/2.303 (7)

In a linear equation, the following formula gives the pseudo-first-order equation

log (q_e_ − q_t_) = log q_e_ − k_1_t/2.303 (8)

Plotting log (q_e_ − q_t_) versus (t) should yield a linear connection between k_1_ and q_e_, which can be evaluated using the slope and intercept.

#### 2.8.2. Pseudo-Second-Order Kinetic Model

The pseudo-second-order equation was written as follows [75]:

dqt/dt = K_2_(q_e_ − q_t_)^2^ (9)

where: K_2_ denotes the second-order rate constant (g mg^−^^1^ min^−^^1^). The following is an example of an integrating equation:

1/(q_e_ − q_t_) = 1/q_e_ + K_2_ (10)

Ho et al. [75] obtain a linear form of the pseudo-second-order equation as follows:

t/q_t_ = 1/K_2_q_e_^2^ + t/q_e_ (11)

Plotting (t/q_t_) versus (t) yields a linear connection, and the slope and intercept may be used to compute the values of the q_e_ and K_2_ parameters, respectively.

#### 2.8.3. The Intraparticle Diffusion Model

The intraparticle diffusion equation [3] is explored as follows:

q_t_ = K_dif_ t^1/2^ + C (12)

where q_t_ (mg g^−^^1^) is the quantities of dye adsorbed at time t. In addition, intercept denotes the value C when the adsorption mechanism follows the intraparticle diffusion process. The values of the intercept provide an idea about the thickness of the boundary layer; i.e., the larger the intercept, the greater the boundary layer effect, and the intraparticle diffusion rate constant, K_dif_ (mg g^−1^ min^−0.5^), is derived using the slope of the regression line.

## 3. Results and Discussion

### 3.1. Characterization of the Adsorbent Material (Binding Mechanism)

#### 3.1.1. FTIR Analysis

**Figure 2:**
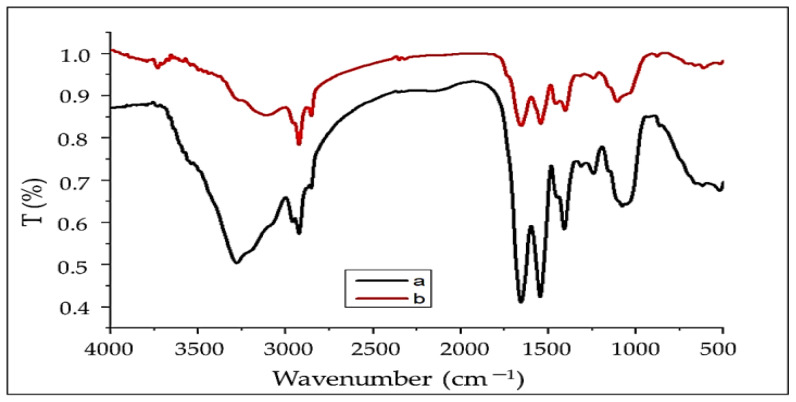
FTIR analysis of *A. platensis* nanoparticle before (a) and after (b) adsorption of MBD.

The FTIR technique was used to investigate the surface of the adsorbent *(A. platensis*) to determine the functional groups responsible for dye adsorption, as each group has a unique energy absorption band. This analysis was carried out on both raw and dye-loaded *A. platensis*. The ability to distinguish characteristic peaks associated with the complex matrix of algae, which incorporates protein, carbohydrates, and lipid fractions, as well as functional groups involved in dye ion adsorption, is a key feature of this approach [76]. In the range of 4000–400 cm^−1^, FTIR spectra of *A. platensis* before and after adsorption were obtained, as shown in Figure 2 and Table 1.

The FTIR spectra of *A. platensis* display the hydroxyl and amine groups’ characteristic absorption bands, which range from 3111 to 3729 cm^−^^1^, for both before and after adsorption, respectively [28]. The band positions at 2923.91 cm^−^^1^ related to the stretching of C–H [14]. Moreover, asymmetric and symmetric stretching of the CH_2_ group is related to the bands at 2923 and 2853 cm^−^^1^, respectively [77]. The carbonyl group of the carboxylic acid is ascribed to the sharp band at 1656 cm^−^^1^ [78,79]. The overlapping amongst broad bands of N–O and N–H with strong peaks for amide has been shown as a single band at 1654 and 1656 cm^−1^ [80]. In addition, ring modes of aromatic rings are ascribed to many small bands (1313, 1317, 1403, and 1409 cm^−^^1^) in the range of 1460–1250 cm^−^^1^ region [81]. The C–N stretch of amide or amine groups can be allocated to the bands at 1242, 1106, and 1079 cm^−^^1^ [62]. C–O stretching vibrations are attributed to the bands at 1079 and 1106 cm^−^^1^ before and after absorption, respectively. P–O, S–O, and aromatic C-H stretching vibrations are responsible for the adsorption bands in the 750–900 cm^−^^1^ range [82]. All of these proposed that the polysaccharide of tested seaweeds may be alginate and fucoidan as reported by Alprol et al. [32]. The transmittance at wave number 3279 cm^−1^ is found to be shifted to 3729 cm^−1^ on adsorption, and this may be responsible for the chemical interaction of the dye with O–H and NH_2_ groups on the *A. platensis* nanoparticles, as reported by Srinivasan and Viraraghavan [15]. The broadening and shifting of peaks on spectra revealed the interaction of functional groups on the surface of the *A. platensis* cell wall with MB dye ions in the aqueous solution. According to the FTIR spectrum of the adsorbent, MBD ions may bind to amino groups and anionic groups due to electrostatic attraction. When the adsorbent was loaded with MBD, the OH, NH_2_, COO, and C=O groups, as well as the aromatic groups were present in the bio-mass, as well as the phosphate and sulfate peak regions. There was a disappearance of the C≡C stretch band at 2143.56 cm^−1^. In addition, there were changes in absorption intensity or shifts in wavenumber of the functional groups as the peaks at 3279, 2959, 2854, 1546, 1409, 1313, 1079, 864 and 661 cm^−1^ were shifted to 3729, 3111, 2853, 1542, 1403, 1317, 1106, 876 and 701 cm^−1^ after MB adsorption, which could be recognized to the contact of ions in the dye with the active sites of adsorbents surface.

#### 3.1.2. UV Examination

**Figure 3:**
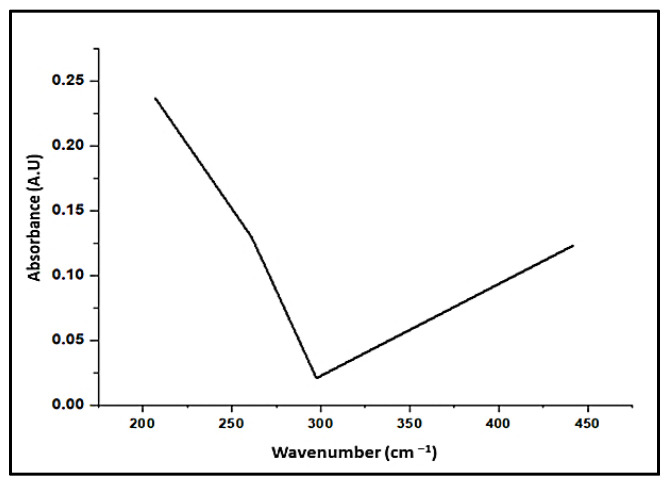
Ultraviolet-visible spectra verified the formation of *A. platensis* nanoparticles.

The optical and structural characterization of adsorbent materials requires the use of UV-vis spectroscopy analysis [77]. The results showed an optimum of 207–412 nm for *A. platensis* nanoparticles as prepared by a hydrothermal process utilizing algae. According to the UV-vis spectrophotometer, there is no distinctive peak change in the reaction mixture. There was no change in peak due to nanoparticle SPR excitations [83]. On nanoparticle *A. platensis*, five peaks occur at 207 and 260 nm with absorbance intensities of 0.236 and 0.130, respectively, as well as 297 nm with an absorbance intensity of 0.021, confirming the formation of hydroxyl groups in the adsorbent material (Figure 3 and Table 2). The spectral bands of flavonoids usually consist of two absorption spectra with maximal values in the regions of 230–290 nm and 300–360 nm [84]. In that case, the existence of phenolic and alkaloid chemicals in the marine algae is revealed by the band appearance at 234–676 nm.

#### 3.1.3. SEM

**Figure 4:**
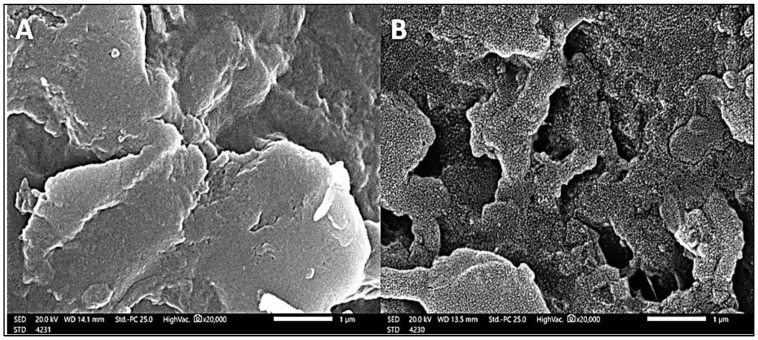
SEM micrographs of *A. platensis* nanoparticles before and after adsorption (**A**,**B**, respectively) of methylene blue.

The surface morphology of *A. platensis* nanoparticles before and after MBD adsorption can be determined using scanning electron microscopy (SEM), as shown in Figure 4. The SEM morphology of *A. platensis* nanoparticles was found to be unique before adsorption, with a nonporous structure and a regular, smooth surface. This revealed that the microalgal cell surfaces were heterogeneous. In addition, before adsorption, SEM analysis revealed that the active sites were homogeneous, resulting in the creation of spherical particles with a uniform distribution. Meanwhile, after MBD adsorption, the cell surface was found to be uneven and porous with aggregation, irregularity, and small particles covering it. In the SEM micrograph of the cells after MBD adsorption, amorphous substances were also aggregated all over the cell surface. Pores of different sizes and shapes could be observed. It is thought that the microscopic particles are cell contents that were discharged during the treatment. The MBD cations covered the surrounding adsorbent particles and occupied the gaps, resulting in the formation of an MB ion monolayer on the surface of the *A. platensis* nanoparticles [85]. Alprol et al. investigated the use of *A. platensis* biomass in the bioremediation of organic dyes from industrial effluents, revealing that changes in morphological state and cell wall matrix for *A. platensis* reflect higher dye surface adsorption. Additionally, Dotto et al. [62] found that the essential components on the surface of the *A. platensis* nanoparticles before adsorption were C (54.0%), N (33.9%), O (9.2%), P (1.8%), and *S. platensis* nanoparticles before adsorption (1.1%). The ratios of C, O, and S increased after the adsorption process, whereas the percentage values of N and P declined. The trapped dye molecules, which include aromatic rings and sulfonic groups, produced this, demonstrating a significant connection between dyes and nanoparticles. Moreover, Dmytryk et al. [86] reported that the amount of all microelements employed in the adsorption procedure, including Co(II), Cu(II), Mn(II), and Zn, increased after adsorption, according to SEM-EDX analysis of *Spirulina* sp. (II).

#### 3.1.4. BET Analysis

**Figure 5:**
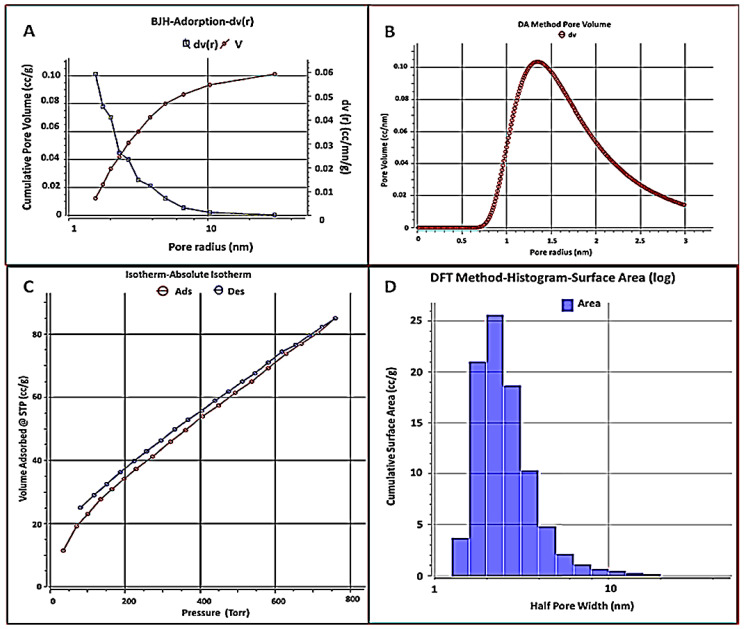
BJH–adsorption–dV(r) at 77 K for *A. platensis* nanoparticles, BJH-Adsorption-dv(r) (**A**), DA method pore volume (**B**), nitrogen adsorption–desorption isotherms (**C**) and DFT method histogram–surface area (**D**).

The physical properties of the adsorbent, as determined by BET analysis, have the greatest influence on the manner of application of the adsorption process. The surface area, size, and distribution of pores are all physical parameters of particles that influence adsorption characteristics by regulating the quantity of adsorbent capacity available and the molecular size that can be adsorbed. The N_2_ sorption isotherms of *A. platensis* nanoparticles were used to investigate the specific surface area, as shown in Table 3. Figure 5 represents the nitrogen adsorption/desorption isotherms. The obtained results are the specific surface area of the A. platensis nanoparticles, which was 139.83 m^2^ g^−^^1^. This is a reasonable value for the synthesized adsorbent. It is worth noting that the larger the surface area, the more efficient the adsorption of MB dye from an aqueous solution. The total volume of pores was 0.131 cc g^−^^1^. While the average particle radius was 9.751 nm, however, the average pore size was 1.88 nm (which indicates mesopore and nanoporous nature), as confirmed by the international union of pure and applied chemistry (IUPAC) classification of pores by size, which showed that pore diameter, D (nm), in the range (2 nm < D < 50 nm, indicates that the type of pore size is mesopore type with characteristic (capillary condensation), while the nanoporous (pores size < 5 nm) and macropore type in scope (D > 50 nm) with characteristic is effectively flat walled [87]. According to the USEPA [88], a nanoparticle, also known as an ultrafine particle, is a small particle of matter with a dimension of 1 to 100 nanometers (nm). Nanoparticles are distinguishable from mesopores by their smaller size, which causes them to have extremely different physical and chemical properties, such as colloidal properties, ultrafast optical effects, and electric properties. Furthermore, the mesopores have the largest effect on the adsorption of organic solutes, allowing solute molecules to access their surfaces. Surface functional groups introduced to the surface of the aggregates as a result of modification changed the adsorbent’s surface character [89]. Li et al. [90] reported that due to their potential to absorb and interact with guest species on their exterior and inner surfaces, as well as in the pore spaces, porous solids, especially mesoporous solids, are attractive materials in many applications. It also contains characteristics that can help materials perform better in terms of energy and power density, lifetime, and stability. Because pores serve as binding or receptor sites during the adsorption phase, this is critical for pollutant trapping [85]. The results demonstrated that *A. platensis* nanoparticles had a significant surface area because algal swelling in aqueous solutions enhances their surface area. This suggests that the former’s adsorptive qualities are greater than the latter’s.

### 3.2. Adsorbent Parameters Optimizations

The goal of the current study was to find the ideal MBD removal parameters, including initial adsorbent biomass concentrations (*A. platensis* nanoparticles), MBD concentration, pH, temperature, and contact time [87].

#### 3.2.1. Effect of Initial MBD Concentration

**Figure 6:**
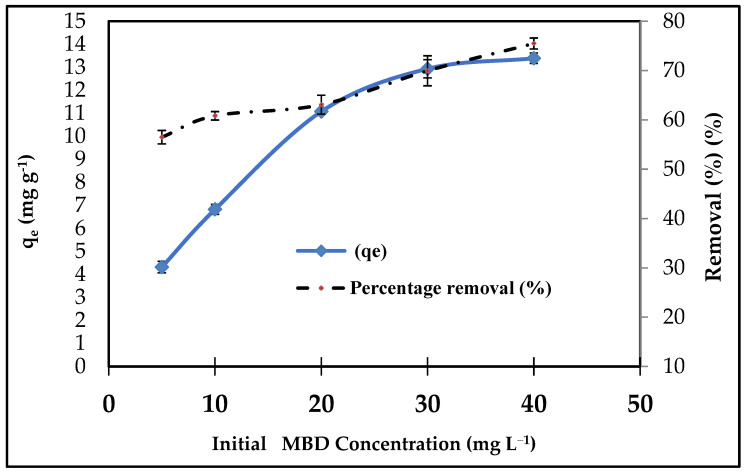
Effect of initial concentration on the percentage removal of MBD onto *A. platensis* nanoparticles (adsorbent amount: 0.1 g; initial volume: 50 mL; pH: 6; contact time: 180 min; 303 K).

A series of tests with varying dye concentrations, namely 5, 10, 20, 30, and 40 mg L^−^^1^, were completed to explore the influence of the beginning MBD concentration on batch operation performance. Figure 6 depicts the influence of the initial MBD concentration on the equilibrium adsorption capacity and removal of nano *A. platensis* at different concentrations. At all studied concentrations, when the initial MBD concentration was increased, qe was augmented [91].

For the lowest starting MBD concentration of 5 mg L^−^^1^, the amount of MBD adsorbed was 4.31 mg g^−^^1^ with a percentage removal of 56.49%, while for the highest initial MBD concentration of 40 mg L^−^^1^, it was 13.39 mg g^−^^1^ with a percentage removal of 75.48%. This result can be explained by the growing driving force that overcomes the MBD’s mass transfer resistance between the aqueous and solid phases [92]. Furthermore, raising the initial dye concentration can improve the sorption process by increasing the number of collisions between MBD cations and adsorbents [93]. For the MBD adsorption, consistent observations were found [94]. In addition, because the initial molar number of pollutant ions reaching the active sites of the adsorbent is higher, the plot slope is steeper in the early stages, and the removal percentage is at its highest [95]. As a result, when the starting dye concentration was higher, there were more ions vying for available sites on the adsorbent’s surface, resulting in a higher MB adsorption capacity [96,97,98].

#### 3.2.2. Adsorbent Dosage

**Figure 7:**
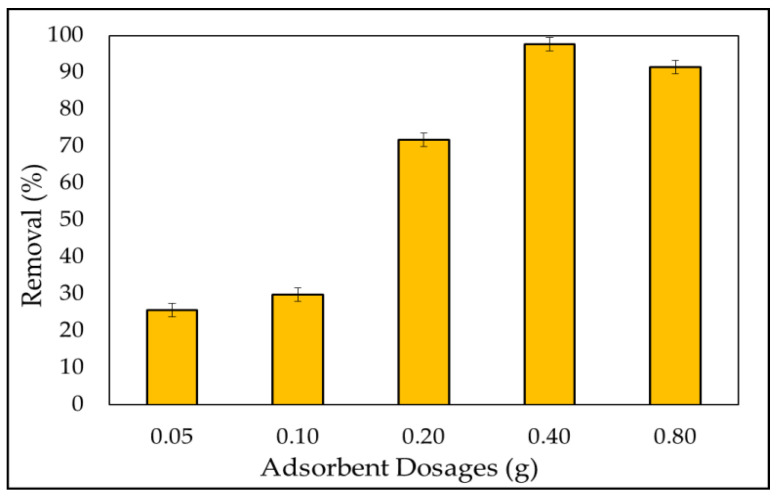
Effect of *A. platensis* nanoparticles adsorbent dosages on the removal of MBD (varying dosage of *A. platensis* nanoparticles from 0.05 to 0.8 g at a dye initial concentration of 5 mg L^−1^; pH value 6; contact time 180 min; initial volume: 50 mL; and 180 rpm).

Examining the amount of adsorbent is useful for selecting the optimum amount of adsorbent for industrial applications from an economic standpoint. As shown in Figure 7, when the dosage was changed from 0.05 to 0.4 g, the dye removal increased, and the largest quantity of dye removal was attained with adsorbent masses of at least 0.4 g, with a percentage removal of 99.4%. It is obvious that when the adsorption dosage is increased, the number of accessible adsorption sites increases, resulting in efficient dye adsorption. As previously noted in multiple articles [28,33,69,99], this is attributable to increases in adsorbent surface areas, which increase the number of adsorption sites available for adsorption. The coagulation of *A. platensis* nanoparticles arose, as a result, resulting in a small surface area and a small reactive site. This phenomenon provides a steady state in the percentage of MB ions adsorbed. However, the uptake decreased at the higher adsorbent dosage (0.8 g). This is due to the partial aggregation of *A. platensis* nanoparticles in solution, which restricts the amount of accessible adsorption sites, as previously reported [100,101]. Therefore, the effective surface area and the total number of binding sites of A. platensis nanoparticles decreases, and the diffusion path length increases [102]. From an economic standpoint, the effect of adsorbent dosage gives a concept of a dye’s ability to be adsorbed with the smallest amount of adsorbent, enabling the recognition of a dye’s capability [103]. In other words, the likelihood of adsorbent–pollutant collisions is enhanced, resulting in improved removal efficiency [104].

#### 3.2.3. Effect of pH

**Figure 8:**
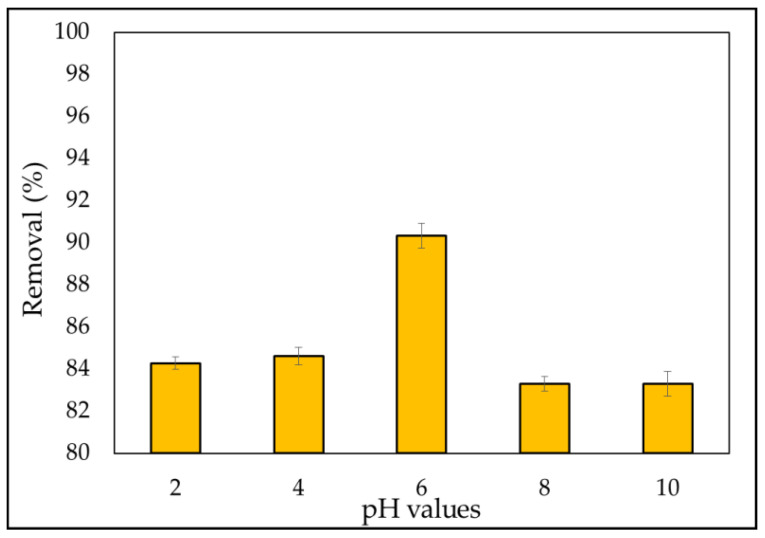
Effect of pH as a function of percentage removal of MBD (varying pH (2–10); dosage of *A. platensis* nanoparticles (0.1 g); MB dye initial concentration 5 mg L^−1^; contact time 180 min; 180 rpm; and initial volume: 50 mL).

The management of the adsorption process was influenced by the pH of the solution. When a basic dye was dissolved, the adsorption of these charged dye groups onto the adsorbent surface was predominantly impacted by the adsorbent’s surface charge, which was influenced by the pH solution. The experiments were conducted at various pH levels ranging from 2 to 10 for 3 h at a constant beginning dye concentration of 5 mg L^−1^, optimum adsorbent dose of 0.1 gm, and contact period of 180 min, with the findings presented in Figure 8. The elimination efficiency was enhanced from 84.2% to 90.3% over the pH range of 2 to 6. MB is a cationic dye that resides in an aqueous solution as positively charged ions.

Furthermore, the low percentage of removal of MB dye at acidic pH (2–4) can be related to the existence of additional H^+^ ions, and the amount of positively charged sites decreases. Meanwhile, due to electrostatic attraction, the number of negatively charged sites grows, favoring MB dye adsorption [105]. The percentage elimination of the MB dyes fell from 90.34% to 83.31% when the pH ranged from 6 to 10. This is due to the formation of a soluble hydroxyl compound between the adsorbent and the MB dye.

During dissolution, MBD occurs, releasing positively charged ions into the solution. The adsorbent surface charge, which is regulated by the solution pH, is the most important factor in cation adsorption onto the adsorbent surface [106]. Furthermore, the results are consistent with those found in the literature [78,107].

#### 3.2.4. Effect of Temperature

**Figure 9:**
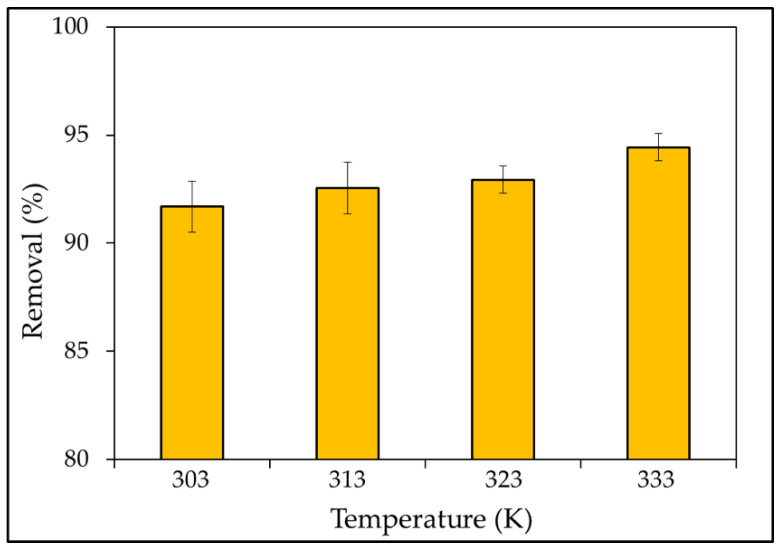
Effect of temperatures function of percentage elimination of MBD (temperatures (303–333 K); adsorbent dose (0.1 g); MB dye initial concentration 5 mg L^−1^; pH value 6; 180 rpm; and initial volume: 50 mL).

The temperature factor must also be managed in adsorption operations. The effect of temperature on removal efficiency was investigated throughout a temperature range of 303 to 333 K, and the results are given in Figure 9. As can be seen, the removal rate of the MBD was highest at 333 K, with a removal rate of 94.4%. Several studies have shown that as the temperature rises above 303 K, the percentage of removal also increases. This could be explained by high temperatures inducing dye molecule diffusion in the interior porous structure of the sorbent [79]. Furthermore, there are two possible explanations for this outcome. At higher temperatures, the pore diameters of adsorbent particles would increase. Due to the breaking of some internal bonds along the edge of the adsorbent’s active surface sites, the number of adsorption sites would increase as well [108]. The effect is stronger at larger concentrations and during endothermic processes, which become more spontaneous as the temperature rises. Abedi et al. [109] reported that as the temperature increases, it causes pollutant ions to separate from the adsorbent surface. As a result, the functional group linkages between pollutant ions and active sites would break down, reducing the pressures between them.

#### 3.2.5. Effect of Contact Times

**Figure 10:**
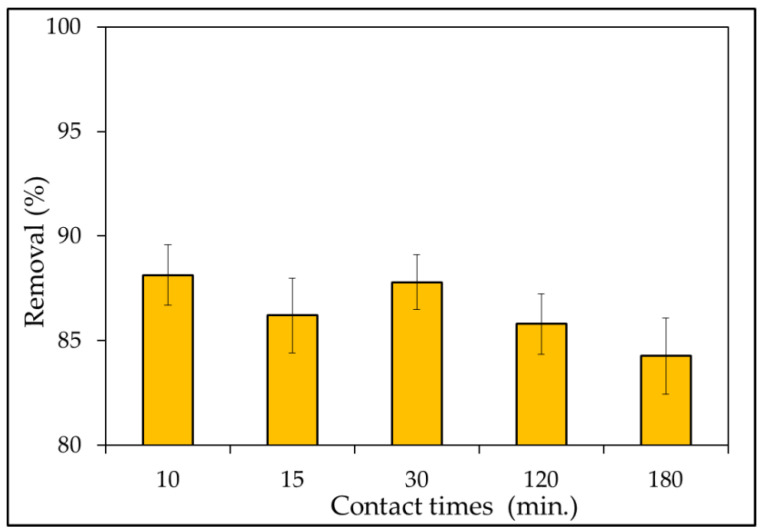
Effect of contact times on the removal (%) of MBD (different contact time (10–180); adsorbent dosage (0.1 g); MBD initial concentration 5 mg L^−1^; pH value 10; temperatures 303 K; 180 rpm; and initial volume: 50 mL).

The effect of contact time on MB dye removal effectiveness was investigated. The adsorption experiments were examined for various contact times (10–180 min) through the addition of a fixed adsorbent dosage 0.1 g in an experiment dye solution of 50 mL at an initial concentration of 5 mg L^−1^ at pH = 10. A rotary shaker was used to agitate the system at a speed of 180 rpm. The findings of the adsorption efficiency versus contact time for the MBD solutions are shown in Figure 10. The rate of removal of MB dye was found to slight decrease gradually from 88.1% to 84.2% in the contact time range of 10–180 min. Within the first 10 min, rapid dye adsorption was seen, with a percentage clearance of 88.1%; then, it slowly slowed until they reached a plateau. Rapid dye adsorption was observed within the first 10 min with a percentage removal of 88.1%; slowly after then, it gradually slowed down until reaching a plateau. The high availability of active sites for dye interaction causes rapid adsorption, which declines with contact time. Additionally, the number of available sites is influenced by kinetic. Furthermore, Idris et al. [96] noted that rapid adsorption at first may be owing to a large number of available surface sites for adsorption, but that after a period of time, the remaining surface sites are difficult to occupy. This is due to the repulsion between the solid and bulk phases’ solute molecules, which takes a long time to reach equilibrium. Additionally, it is mostly due to active site saturation, which prevents further adsorption [110].

However, the rate of dye removal slows until equilibrium is reached after 3 h, when the amount of dye molecules occupying active sites increases [78]. According to Peng et al. [111], adsorption by adsorbents seems to be mostly dependent on the porous structure; hence, adsorbents take time to diffuse through pores. Moreover, Afroze and Sen [112] noted that there are more free binding sites available at first, but as the number of available sites for binding metal ions on the surface declines, the number of available sites begins to decrease and stagnate [112].

### 3.3. Mechanism of Adsorption

As demonstrated by FTIR analysis, the adsorption of MB dye from an aqueous solution by *A. platensis* nanoparticles is strongly dependent on the numerous functional groups on the adsorbent’s surface, such as hydroxyl, carbonyl, NH_2_, aromatics, etc. After protonation and deprotonation, the functional groups on the surface of *A. platensis* nanoparticles can be charged (negative and positive) or neutral. The following are examples of a hypothetical adsorption mechanism: (i) under acidic conditions, hydrogen atoms (H^+^) in the solution can protonate the amine and hydroxyl groups of *A. platensis* nanoparticles; (ii) the carboxylic groups are deprotonated, resulting in negatively charged carboxylate ligands (–COO^−^) binding the charged MB. In addition, (iii) the electrostatic and hydrogen-bonding interactions can be established between the surface hydrogens of the hydroxyl groups (H–donors) on the *A. platensis* nanoparticles surface and the nitrogen atoms (H–acceptors) on the MB surface. Dipole–dipole hydrogen bonding is another label for this phenomenon. The n–π interactions develop between electron donor atoms with pairs of electrons, such as oxygen or nitrogen, and aromatic rings as acceptors. Furthermore, oxygen in the carbonyl groups on the adsorbent’s surface works as an electron donor in this study, while the aromatic rings of MB act as electron acceptors. These results are comparable to Tran et al. and Salazar-Rabago et al. [113]. According to Singh and Singh [114], the reducing agent potential of various algal aqueous extracts could cause extracellular nanoparticle synthesis. It has the required potential because of the presence of proteins, polysaccharides, reducing sugar, pigments, and other compounds that can activate metal ion reduction on pollutants and then precipitate as nanoparticles. While in the case of intracellular production, the ability to lower the ionic pollutant component is due to several factors such as algal metabolism, which includes respiration and photosynthesis, which might be advantageous in a reduction circumstance.

### 3.4. Adsorption Dynamics

#### 3.4.1. Kinetic Study

**Figure 11:**
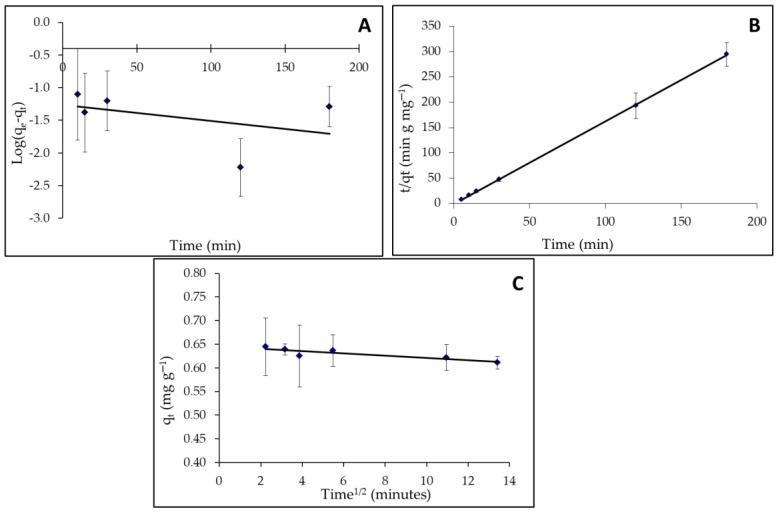
Adsorption kinetic models for MB on *A. platensis* nanoparticles; (**A**): pseudo-first-order; (**B**): pseudo-second-order; and (**C**) intra-particle diffusion plots.

Adsorption is the interaction of an adsorbent (a solid phase) with a contaminated aqueous solution. This process can go on indefinitely until equilibrium is reached, which refers to the amount of pollutant adsorbed and the proportion remaining in the solution at a constant concentration [115]. It is vital to be able to forecast the rate at which contaminants are eliminated from an aqueous solution when planning an adsorption water treatment facility. The kinetics of MBD solute adsorption data was studied in terms of pseudo-first, second-order, and intra-particle diffusion mechanisms at optimum conditions to learn more about the adsorption mechanism and potential rate-controlling phases including mass transfer and chemical reaction (as presented in Figure 11A, B, and C, respectively). Using the slope of the linear plots of ln(q_e_ − q_t_) against t for each solute, the rate constant (K_1_) for MBD was calculated using the pseudo-first-order rate formula (Equation (8)). The slope and intercept of plots of t/qt against t were used to derive the pseudo-second-order rate constant (K2) using Equation (11). The slope of plotting Equation (12) was used to find the K_dif_ of the intra-particle model. The correlation coefficients agreed with experimental data and model predicted values (R^2^, values close or equal to 1 [116]). The model’s R^2^ value is relatively high, indicating that it accurately represents the kinetics of MBD adsorption. According to observed results, the experimental data are followed by pseudo-second-order and the values of the rate constants with the appropriate correlation, as shown in Table 4. R^2^ values for pseudo-second-order models are quite high (>0.999) when compared to pseudo-first-order (Figure 11) and intra-particles diffusion models. These findings imply that this model accurately represents the kinetics of MB adsorption on microalgae and that the process is referred to as chemical sorption. Furthermore, the pseudo-second-order kinetic model’s computed values of qe are substantially closer to the experimental values of qe than the pseudo-first-order model’s. The pseudo-first-order model relates the number of adsorption sites on the adsorbent surface occupied by pollutant particles to the number of vacant sites [117]. Low regression coefficients (R^2^), on the other hand, indicate that the model does not well fit the experimental data and demonstrates that intra-particle diffusion is not driving the adsorption process.

#### 3.4.2. Adsorption Isotherm Modeling

**Figure 12:**
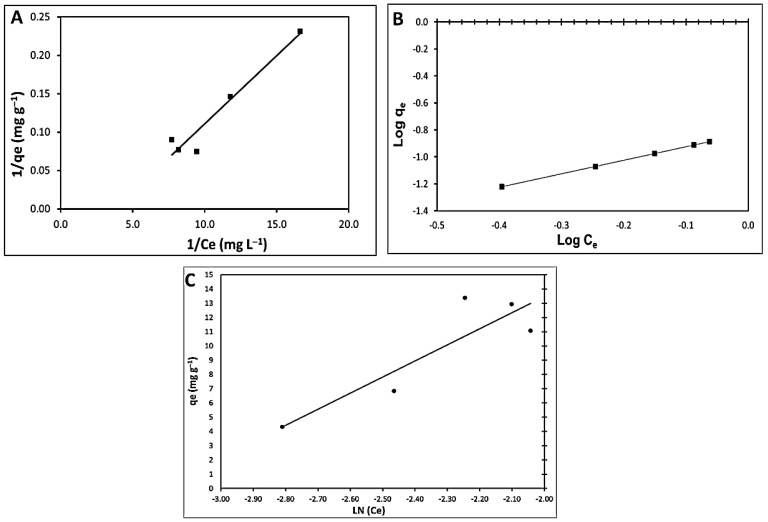
Linear isotherms of MBD adsorbed on *A. platensis* nanoparticles; (**A**) Langmuir; (**B**) Freundlich; and (**C**) Tempkin isotherm.

The Langmuir, Freundlich, and Tempkin adsorption isotherms of the nano-adsorbents were employed in nonlinear analysis to investigate the relationship between non-absorbent adsorption capacity and dye ion concentration, as shown in Figure 12A–C.

Table 5 lists the values of the associated isotherm variables, their correlation coefficients (R^2^), and related standard errors (S.E.). In comparison to the Tempkin isotherm model (R^2^ > 0.790), the results demonstrated that fitting experimental data into the Freundlich isotherm model and Langmuir isotherm model (R^2^ > 0.936) yielded high R^2^. These results demonstrate that the Freundlich isotherm model can provide a good fit to the experimental data, implying that the Freundlich adsorption system modeling is appropriate. Maximum adsorption corresponds to a saturated monolayer of adsorbate molecules on the adsorbent surface, and the energy of adsorption is constant. The highest adsorption capacity found using the Langmuir model was 58.8 mg^−1^, as indicated. The adsorption constant ‘b’ is connected to the affinity of binding sites (L g^−1^), and a lower value of ‘b’ (3.8) indicates that the adsorbent particles’ radius was tiny when it came to adsorption. The dimensionless separation factor, R_L_, was utilized to forecast the affinity of the nano-adsorbent surfaces toward the MBD ions using the Langmuir parameters, which are given in the following equation:

R_L_ = 1/(1 + bC_i_) (13)

Because the values of R_L_ (0.127) are between 0 and 1, MBD adsorption on a nano-adsorbent appears to be favorable [118]. On the other hand, K_F_ is a Freundlich constant that represents the adsorption capacity on heterogeneous sites with non-uniform energy level distribution, and n represents the intensity between adsorbate and adsorbent [119]. The value of K_F_ was found to be 6.66 as the plot yielded. These findings indicate that this model accurately represents that the isotherm of the MBD adsorption process on the adsorbent is a chemical sorption process. When 1/n is less than 1, chemical adsorption occurs; however, when 1/n is more than 1, cooperative adsorption occurs, which is more physically advantageous and involves strong interactions between adsorbate particles.

Furthermore, the value of the factor “1/n” in this study is greater than 1, indicating that employing this isotherm equation to execute the chemical sorption mechanism on an outside surface is preferable. Values of n > 1 for MB particles show a heterogeneous nature of sorption and positive binding [113]. Ion exchange, chelation, and complexation are referred to as chemisorption, whereas electrostatic interactions and van Waals forces are referred to as physisorption [120]. In chemisorption processes [121], functional groups on the adsorbent surface are critical for binding MB dye ions. The electrostatic interactions between the negatively charged cell walls of the adsorbent and the cations in the solution are then provided via ion exchange [122]. The Tempkin sorption isotherm model was preferred to investigate the adsorption abilities of the adsorbent for MBD. The observed amounts corresponding to the adsorption isotherm plateau are all lower than the theoretical monolayer saturation capacity in the Tempkin isotherm model produced using nonlinear regression, showing that the Tempkin isotherm modeling for the adsorption system is unsuitable, as shown in Table 5. Chojnacka et al. [123] studied the equilibrium process of blue-green algae *Spirulina* sp., which confirmed that the adsorptive surface depended on the morphological form of microalgae. Both autotrophic and lyophilized biomass had the highest surface area and the highest adsorption capacity. The adsorption capacity is thus proportional to total bio-sorption capacity. The geometrical surface area of cells is the sum of the adsorptive surface (3.7% of the total geometrical surface that has the affinity to bind methylene blue by physical adsorption) and another surface on which there are found places with high affinity to metal ions. This means that the sorptive surface area of cells that are different morphologically differs, but the quality of this surface is the same. This indicated that the nature of the cell wall of microalgae is the same, but the surface is developed (packed) differently in the unit mass of the sorbent.

### 3.5. Goodness of Model Fit

The Chi-square statistic and the coefficient of determination (R^2^) for linearized data were employed to assess the fit goodness of the applied mathematical equations to the experimental data [124]. In addition, the isotherm constants were calculated using a nonlinear regression basin’s percentage error function and compared to the less precise linearized analytical values (R^2^ values) [125].

Chi-square error equation is calculated as follows [94]:

(14) $$X^{2}=\sum_{i=1}^{N}{\left|\frac{(q_{e,\exp,\mathrm{isotherm}}-q_{e,\mathrm{calc}})\times 2}{q_{e,\exp,\mathrm{isotherm}}}\right|}_{i}$$

where q_e,exp_ is the experimental value, q_e,cal_ is the calculated value, m denotes the number of observations, and n is the number of data points in the experiment. The Langmuir isotherm model was determined to have the best fit of the MBD adsorption onto *A. platensis* nanoparticles when the error percentage values were compared, as shown in Table 4. The model has a low error percentage value and a high correlation coefficient. Meanwhile, the Chi-square error values found in the Freundlich isotherm model are significantly higher than those obtained in the other two models.

Table 6 shows the capacity of *A. platensis* nanoparticles and various functionalized adsorbents to remove MB dye. It was observed that due to the presence of functional groups on the surface of *A. platensis*, this adsorbent outperforms its peers in terms of its ability to remove a high percentage (97%) and capacity (58.82 mg g^−1^) of the pollutants exposed to it in a short amount of time, verifying the novelty and importance of the prepared adsorbent in the current study among the other listed adsorbents.

### 3.6. Reuse of the Adsorbent

**Figure 13:**
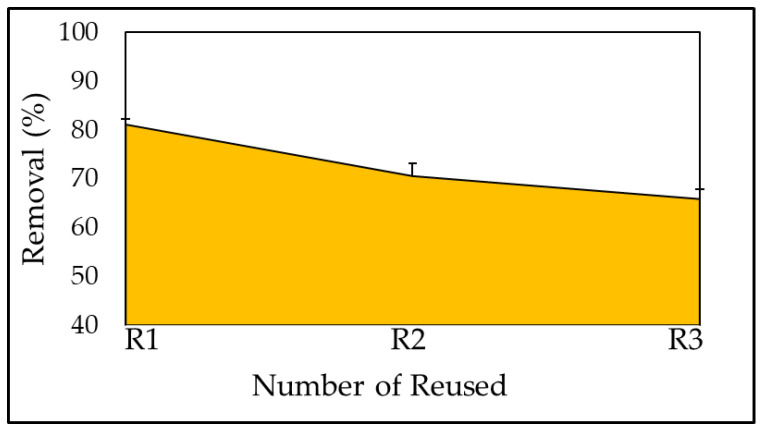
Reused of the adsorbent for removal of MBD.

The regeneration of dye ions from biomass produced after biotreatment is a crucial factor in the regeneration of the adsorbent to reduce the process cost and ensure the adsorbent’s continuous supply. The sorbent was employed in both its newly synthesized and reused forms following dye adsorption from the loaded sorbent (the regenerated sorbent may be reused many times). Because the *A. platensis* nanoparticle is not a readily available substance, it must be regenerated. The efficiency of the adsorption cycle was demonstrated in Figure 13. At pH 6 and 30 °C for 180 min, MB dye solution (0.1 g) was combined with *A. platensis* nanoparticles for regeneration experiments. To estimate adsorbed dye on the adsorbent, the remaining dye concentration in the solution was measured. After that, the adsorbent was filtered and washed to remove the adsorbed dye, which was then dried in a vacuum oven at 50 °C for 24 h. At 10 mg L^−1^ of MB, dye-loaded adsorbent was allowed to come into contact with 50 mL of distilled water in a 100 mL conical flask, which was stirred at 150 rpm on the shaker for 180 min. A spectro-photometer was used to determine the amount of dye desorbed. A batch test revealed that the adsorbent can remove up to 70.5% of the MBD after the first round, which reduced slightly to 70.55% and 65.86%, after the second and third cycles. According to the plot, adsorption values were higher than regeneration analysis values, indicating that the adsorbed MBD dropped during the third phase as the amount of biomass in the solution and the number of accessible sites for the adsorption process decreased. The ability of each biomass-based adsorbent to function well in an adsorption process would decrease the time until it reached an irreversible phase after repeated adsorption cycles. Once a saturated bio-adsorbent has operated unreliably for further use due to an extreme decline in its adsorption capacity, it must be disposed of and managed correctly in accordance with relevant parties’ requirements, and it must be labeled as hazardous waste [141]. Regeneration investigations displayed that it is possible to fully eliminate the ions of MBD bound with the biomass and to regenerate the adsorbent allowing its successive use [123].

## 4. Conclusions

This study measured the effect of initial pH, biomass amounts, temperature, contact time, and dye concentrations on the adsorption of MBD ions by *Arthrospira platensis* NIOF17/003 nanoparticles as green synthesis. Batch operations were used to conduct adsorption kinetic and equilibrium experiments. Langmuir isotherm, Freundlich, and Tempkin were used to analyze the adsorption data acquired under ideal conditions. The Freundlich isotherm with R^2^ = 1 accurately depicted the equilibrium process. In addition, the outcomes for the pseudo-second-order kinetic model indicated a high correlation coefficient. According to the adsorption kinetic estimates, the MBD ion removal rate was at its maximum at the start of the procedure. The highest percentage removal (99%) of MBD from aqueous solution by nanoparticles of *A. platensis* was discovered under the following ideal conditions: an initial MBD concentration of 40 mg L^−1^, temperature 333 K, pH 6, adsorbent 0.4 g, and equilibrium state reached after 15 min of agitation. In addition, FTIR, SEM, UV, particle size, pore volume, and pore diameter were used to analyze the adsorption process of the adsorbent. The FTIR spectrum revealed that carboxyl and carbonyl were the main functional groups involved in the sorption process. The sorption equilibrium research confirms a chemical process for dye removal on nano-adsorbents. The substance can be reused in future dye sorption–desorption cycles after the residual dyes have been desorbed, and the sorptive capacity has been kept within noteworthy high limits. Since *A. platensis* is a low-cost biomass with significantly high adsorption ability at low concentrations, biomass is an alternative adsorbent for the treatment of wastewater MBD ions.

## Figures and Tables

**Table 1:** Wavenumber and functional groups of FTIR spectroscopy investigation of *A. platensis* nanoparticles before and after sorption of MBD.

Before Adsorption		After Adsorption		
Wave Number (cm^−1^)	Annotations	Wave Number (cm^−1^)	Difference (cm^−1^)	Annotations
3279.90	O–H group	3729.92	450	O–H group and –NH groups
		3111.91	new peaks	
2959.97 & 2923.44	CH_2_ group	2923.44	36.5	Aliphatic C–H group
2854.48	Asymmetric CH_3_ & symmetric CH_2_ stretching	2853.86	0.61	Asymmetric CH_3_ and symmetric CH_2_ stretching
2143.56	C=O of the carboxylic groups or ester groups	disappears		C=O of the carboxylic groups or ester groups
1656.51	C=O, C=N, N–H or C=C groups	1654.92	1.58	C=O, C=N, N–H or C=C groups
1546.33	C=C stretching	1542.98	3.34	C=C stretching
		1456.35	new peaks	C=C stretch aromatic
1409.30	–C=O stretches	1403.10	6.19	Sp3 C–H bend
1313.08	C–O	1317.83	4.75	
1242.68	–O–C links of the organic phosphate groups	1242.26	0.41	C–N stretch of amide or amine groups
		1106.10	new peaks	
1079.08	C–O stretching of ether groups	disappears		
864.69	P–O, S–O, and aromatic C–H stretching	876.95	12.258	P–O, S–O, and aromatic C–H stretching
		701.65	new peaks	
661.77	–P–O, –S–O, and aromatic –CH stretching or Silicate	659.96	45.33	Aromatic sp2 C–H bend or Alkene sp2 C–H bend
621.32		616.44		
522.32		519.50	2.815	
474.68		464.72	9.96	
		420.83		

**Table 2:** UV-visible spectrum of methanolic extract of *A. platensis* nanoparticles.

Peak No.	Wavelength (nm)	Optical Density (O.D)
1	441.5	0.123
2	297.5	0.021
3	269.5	0.105
4	261	0.13
5	207	0.237

**Table 3:** BET analysis of *A. platensis* nanoparticles.

Characteristics	Data	Unit
Specific surface area (Multipoint)	139.837	m^2^ g^−1^
Langmuir method	261.836	m^2^ g^−1^
BJH adsorption	71.0792	m^2^ g^−1^
BJH desorption	60.5881	m^2^ g^−1^
Total pore volume (Vp)	0.131752	cc g^−1^
Mean pore size	1.88438	nm
Average particle radius	9.751	nm

**Table 4:** Adsorption kinetic model rate constants for MB on *A. platensis* nanoparticles.

Model	Parameter	Value
Pseudo-First-Order Kinetic	q_e_ (exp.)	0.626
	q_e_ (calc.) (mg g^−1^)	19.45
	k_1_ × 10^3^ (min^−1^)	5.76
	R^2^	0.169
Pseudo-Second-Order Kinetic	q_e_ (calc.)	0.61
	k_2_ × 10^3^ (g mg^−1^ min^−1^)	2283.97
	R^2^	0.999
Intra-Particle Diffusion	C (mg g^−1^)	0.0024
	K_dif_ (mg g^−1^ min^1/2^)	0.645
	R^2^	0.775

**Table 5:** Factors of isotherm models and error functions.

Isotherm Model	Isotherm Parameter	Value	X^2^
Langmuir	Q_max_ (mg g^−1^)	58.82	11.13
	R_L_	0.127	
	b	3.8	
	R^2^	0.936	
Freundlich	n	0.595	822.1
	1/n	1.682	
	K_F_ (mg^1−1/n^L^1/n^g^−1^)	6.66	
	R^2^	1	
Tempkin	A (L g^−1^)	24.337	233.45
	B (mg L^−1^)	36.091	
	R^2^	0.790	

**Table 6:** Comparison of the maximal uptake capacity of MBD with other adsorbents.

Adsorbent	Capacity (mg g^−1^)	Conditions	Ref.
*Eugenia umbelliflora*	157.2	pH 10, sorbent dosage 6 g L^−1^, and time 35 min, respectively	[126]
MWCNT–SH	166.7	60 (min) 298 K pH 6	[127]
MWCNT	100	60 (min) 298 K pH 6	[127]
Carbon nanotubes	35.4	45 (min) 273 K pH 7	[128]
Composite of graphene–CNT	65.79	30 (min) 283 K pH 7	[129]
The brown alga	38.61		[130]
Modified saw dust	111.46		[131]
Spent rice biomass	8.3		[132]
Banana peel	20.8	pH 7.2	[133]
*Artocarpus odoratissimus* skin	184.6	pH 4.6	[134]
Polydopamine microspheres	161.29	298 K	[135]
Poly-melamine-formaldehyde polymer	80.8	298 K	[136]
Ho-CaWO_4_ nanoparticles	103.09	pH 2.03, time 15.16 min, adsorbent dosage 1.91 g	[137]
Steam-activated carbon produced from lantana camara Stem	19.84	20 °C, 60 min, 2 g, 50 mg L^−1^, pH 8.	[98]
Activated carbon/ureaformaldehyde composite	1.414	T = 298 K; MB Conc. = 5 mg L^−1^; Shaking speed = 100 rpm; Natural pH = 6.4	[138]
Activated carbon coated with zinc oxide (ZnO nanoparticles)	66.66	MB Conc. = 5 mg L^−1^, contact time = 120 min and AC–ZnO conc. = 1.5 g/L	[107]
MWCNTs/Gly/β-CD	90.90	20 min; MB Conc. = 5 mg L^−1^	[139]
*Sargassum latifolium*	7.8	MB Conc. = 5 mg L^−1^; 120 min; temperature of 50 °C	[6]
Hydrolyzed wheat straw	5.8	MB Conc. = 5 mg L^−1^; 5 to 15 min	[140]

## Data Availability

The data that support the findings of this study are available from the authors upon reasonable request.
